# Effects of Soil Conditioner (Volcanic Ash) on Yield Quality and Rhizosphere Soil Characteristics of Melon

**DOI:** 10.3390/plants13131787

**Published:** 2024-06-27

**Authors:** Dongxu Xue, Yangyang Wang, Hong Sun, Lina Fu, Lihe Zhu, Jiaqi Liu, Zhenyi Zhi, Jiayi He, Wei Wang, Chunyan Wu

**Affiliations:** 1 College of Horticulture, Jilin Agricultural University, Changchun 130118, China; x2693963339@163.com (D.X.); yy11220718@163.com (Y.W.); fulina_2023@163.com (L.F.); m18443995528@163.com (L.Z.); 13930484161@163.com (J.L.); 15891072494@163.com (Z.Z.); h13019260277@163.com (J.H.); 2 College of Mechanical Science and Engineering, Jilin University, Changchun 130022, China; sunhongnjy@163.com

**Keywords:** melon, quality, rhizosphere soil, soil conditioner, soil characteristics, yield

## Abstract

In this study, the effects of soil conditioners on the growth and development of melons and the rhizosphere soil environment were explored. The optimal amount of added soil conditioner was screened to solve the practical production problems of high-quality and high-yield thin-skinned melon. The melon variety “Da Shetou” was used as the material. Under the conditions of conventional fertilization and cultivation technology management, different soil conditioners were set up for potted melons. The effects of Pastoral soil (CK), 95% Pastoral soil + 5% volcanic ash soil conditioner (KT1), 85% Pastoral soil + 15% volcanic ash soil conditioner (KT2), 75% Pastoral soil + 25% volcanic ash soil conditioner (KT3), 65% Pastoral soil + 35% volcanic ash soil conditioner (KT4), and 55% Pastoral soil + 45% volcanic ash soil conditioner (KT5) on melon yield, quality, and rhizosphere soil characteristics were investigated. The soil microbial community was analyzed using Illumina MiSeq technology. Compared to CK, KT1, KT3, KT4, and KT5, the KT2 treatment could improve the single fruit yield of melon, increasing it by 4.35%, 2.48%, 2.31%, 5.92%, and 2.92%. Meanwhile, the highest contents of soluble protein, soluble solid, and soluble sugar in the KT2 treatment were 1.89 mg·100 g^−1^, 16.35%, and 46.44 mg·g^−1^, which were significantly higher than those in the control treatment. The contents of organic matter, total nitrogen, alkali-soluble nitrogen, nitrate nitrogen, ammonium nitrogen, available potassium, and available phosphorus in melon rhizosphere soil were the highest in the KT2 treatment. Through Alpha diversity analysis, it was found that the Chao1 index, Shannon index, and ACE index were significantly higher in the KT1 treatment than in the control, while, among all groups, the Simpson index and coverage were not significantly different. The dominant bacteria in the six treated samples were mainly *Actinobacteriota*, *Proteobacteria*, *Cyanobacteria*, *Chloroflexi*, *Acidobacteria*, *Bacteroidetes*, *Myxomycota*, *Firmicutes*, *Gemmatimonadota*, *Verrucomicrobia*, and *Planctomycetes*, which accounted for 96.59~97.63% of the relative abundance of all bacterial groups. Through redundancy analysis (RDA), it was found that the organic matter, electrical conductivity, available phosphorus, and nitrate nitrogen of melon rhizosphere soil were the dominant factors of bacterial community change at the dominant genus level. In summary, 15% ash soil conditioner applied on melon was the selected treatment to provide a theoretical reference for the application of soil conditioner in facility cultivation.

## 1. Introduction

Melon (*Cucumis melo* L.), an annual vine herb in the cucurbit family, is an important and efficient horticultural crop in China [1]. Due to the application of unreasonable cultivation techniques, such as the excessive use of chemical fertilizers and pesticides, soil salinization, soil nutrient loss, fertilizer utilization rate reduction, and micro-ecological environment imbalance occur frequently in melon soil, which seriously restrict the development process of the intensification, scale, and quality of the melon industry [2,3].

Soil conditioner is composed of natural polymers such as polysaccharides, polycyclic viscous organic compounds, resin gums, and humic acids obtained by the pyrolysis, distillation, and concentration of peat, melon bean extract, lignite, gum, and pulp waste liquid. Its main functions are improving soil structure, retaining soil moisture, improving soil salinization, and eliminating heavy metal pollution [4,5,6].

Studies have shown that soil conditioner can promote the growth and development of vegetable crops such as rape, celery, tomato, mustard, potato, and cucumber; increase the yield of tomato, ginger, leek, and other vegetables; enhance the contents of soluble solid, protein, total sugar, and vitamin C in vegetables; and increase the contents of soil organic matter, available phosphorus, available potassium, and alkali-hydrolyzed nitrogen. At the same time, it can effectively improve the physical and chemical properties of soil and repair heavy metal pollution [7,8,9,10].

In recent years, a lot of studies have found that different types of soil conditioners affect the growth and development of vegetable crops by changing the number and community structure of microorganisms. For example, in tomato soil treated with *Rhodobacter* genus YH-07 inoculation with organic fertilizer, the abundance of Bacillus, *Altererythrobacter*, *Cryptococcus*, and *Saprospiraceae* increased, while the abundance of *Chryseolinea* and *Fusarium* decreased [11,12]. Biochar from vegetable straw could increase the relative abundance of *Actinomycetes*, *Proteobacteria*, *Oleochytria*, and *Rozobacteria* in cucumber continuous cropping soil. The Shannon index, ACE index, and Chao1 index first increased and then decreased with the increase in the amount of biochar. Compared with conventional fertilization treatment, an increased application of soil additives leads to an increase in the community and diversity of fungi and bacteria in the rhizosphere of Chinese cabbage, with a relative abundance of four dominant bacteria phyla as follows: *actinomycetes* (2.15%), *chlorocurvula* (27.55%), *blastomonas* (13.83%), and *Bacteroides* (60.22%). Additionally, a decrease in penicillium was observed in fruit and vegetable pathogens [13]. The addition of oyster calcium soil conditioner in continuous cropping soil increased the Shannon, Simpson, ACE, and Chao1 indexes of Alpha diversity and also increased the abundance of five bacterial phyla, including Proteus, Firmicutes, and Bacteroides. The abundance of *ascomycetes* and *basidiomycetes* in fungi was reduced, and the continuous cropping of fungal soil resulted in its transformation into bacterial soil, thus alleviating the obstacles of continuous cropping [14].

In this study, the melon variety “Da Shetou” was used as the experimental material, and different soil conditioner ratios were applied to potted melon. The yield and fruit quality of melon, as well as the physicochemical properties of rhizosphere soil, were investigated, and the soil microbial community was analyzed using Illumina MiSeq technology (Illumina Inc., San Diego, CA, USA) to determine the effects of different soil conditioner ratios on the yield, quality, and rhizosphere soil characteristics of melon. The effects of soil conditioner on the growth and development of melon and the rhizosphere soil environment were discussed. The optimum soil conditioner ratio was selected to provide a theoretical reference for its application in facility cultivation to solve the practical production problem.

## 2. Results

### 2.1. Effect of Different Soil Conditioner Ratios on Yield of Melon

The effects of different soil conditioner ratios on melon yield are shown in Table 1. Under the experimental conditions, only the KT2 treatment significantly increased the single fruit yield of melon. Compared to the CK, KT1, KT3, KT4, and KT5 treatments, the KT2 treatment significantly increased single fruit yield by 4.35%, 2.48%, 2.31%, 5.92%, and 2.92%, respectively.

As concerns total yield, the KT1, KT2, and KT3 treatments caused it to significantly increase. Compared to CK, the total yield increased by 13.00%, 16.24%, and 14.06%.

### 2.2. Effect of Different Soil Conditioner Ratios on Fruit Quality of Melon

The contents of soluble proteins, solid matter, and sugars in melon fruits treated with KT3 were the highest among all groups (Table 2); in particular, the content of soluble protein was significantly increased by 8.62%, 5.59%, 9.88%, and 6.78% compared to CK, KT1, KT4, and KT5 treatments. The amount of soluble solids in the KT3 treatment increased significantly by 10.32%, 9.36%, 2.89%, 17.20%, and 9.88% compared to that of the CK, KT1, KT2, KT4, and KT5 treatments. The soluble sugar content was also significantly increased in the KT3 treatment group by 8.96%, 10.10%, and 12.91% compared to the CK, KT4, and KT5 treatments. The content of vitamin C in melons treated with KT2 was the highest, significantly increasing by 16.49%, 15.67%, 14.60%, 27.64%, and 12.86% compared to CK, KT1, KT3, KT4, and KT5 treatments. The soluble solids and vitamin C contents of melon fruit were the lowest in the KT4 treatment, being significantly reduced by 6.24% and 8.74% compared to CK.

In addition, the organic acid content of melon fruit was significantly increased by different soil conditioner ratios in the rhizosphere of melon in the following order: KT5 > KT4 > KT3 > KT2 > KT1. Among them, KT5-treated melon fruits had the highest organic acid content.

The KT2 treatment significantly increased the solid acid ratio of melon fruit compared to CK, KT1, KT3, KT4, and KT5 treatments, increasing by 5.97%, 6.32%, 4.04%, 21.90%, and 18.79%. The KT4 and KT5 treatments significantly reduced the solid acid ratio of melon fruit, with the lowest being in the KT4 treatment, which was 13.07% lower than CK.

### 2.3. Effects of Different Proportions of Soil Conditioner on Physical and Chemical Properties of Melon Rhizosphere Soil

#### 2.3.1. Effects of Different Soil Physical and Chemical Conditioners on Soil Physical and Chemical Indicators

With the increase in soil regulator ratio, the soil pH gradually increased. Among them, the KT2, KT3, KT4, and KT5 treatments all reached a significant level of 5%, compared to CK. In addition, both the KT2 and KT3 treatments significantly reduced the electrical conductivity of melon rhizosphere soil (Table 3).

The addition of different amounts of soil conditioners had an effect on the bulk density of melon rhizosphere soil. Compared with CK, the KT1, KT2, KT3, KT4, and KT5 treatments decreased by 3.24%, 7.45%, 10.49%, 13.08%, and 16.11%, respectively. The bulk density of the soil showed a decrease with the increase in the amount of soil conditioner, and the KT5 treatment had the largest decrease compared with the control treatment. In addition, the KT1, KT2, KT3, and KT5 treatments significantly increased the content of rhizosphere soil organic matter by 11.58%, 20.81%, 16.61%, and 6.97% compared to the control.

Soil is the basic environment for the survival and development of organisms; the physical and chemical properties of soil directly affect its growth, reproduction, and the metabolic functions of its organisms [15]. In terms of soil nitrogen, except for the KT4 treatment, the other treatments increased the contents of total nitrogen, alkali-hydrolyzed nitrogen, nitrate nitrogen, and ammonium nitrogen in melon rhizosphere soil; in particular, the KT1, KT2, and KT3 treatments significantly increased the contents of the latter two. In addition, KT2 and KT3 treatments significantly increased the contents of available potassium and available phosphorus in the rhizosphere soil of melon, while KT1, KT4, and KT5 showed no significant changes compared to CK.

#### 2.3.2. Analysis of Bacterial Composition and Relative Abundance in Rhizosphere Soil of Melon with Different Soil Conditioner Ratios

**Figure 1:**
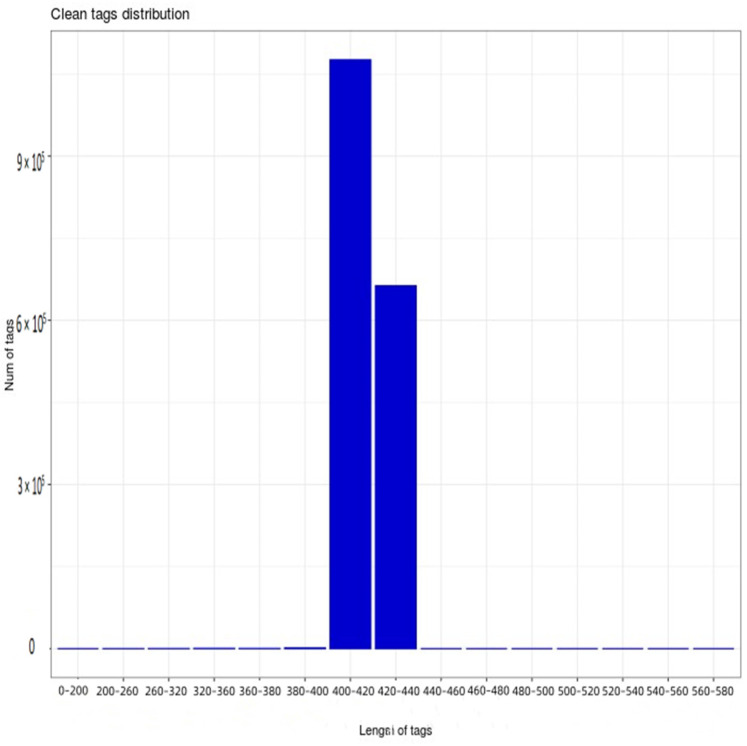
Distribution map of high–quality sample sequences.

Furthermore, 16s rRNA gene sequencing was performed on 18 samples from six treatments. After the further removal of chimeras and short sequences, a total of 1,741,628 high-quality sequences were obtained, the length of which was mainly 400–440 bp (Figure 1), with 1,076,075 high-quality sequences being 400–420 bp, while 663,179 were 420–440 bp. According to Figure 1, the dilution curve of each sample shows a relatively stable trend, indicating that the sequencing data are reasonable. At the same time, this indicates that the sequencing depth is sufficient to cover a wide range of bacterial types, so that subsequent bacterial community analysis can continue.

**Figure 2:**
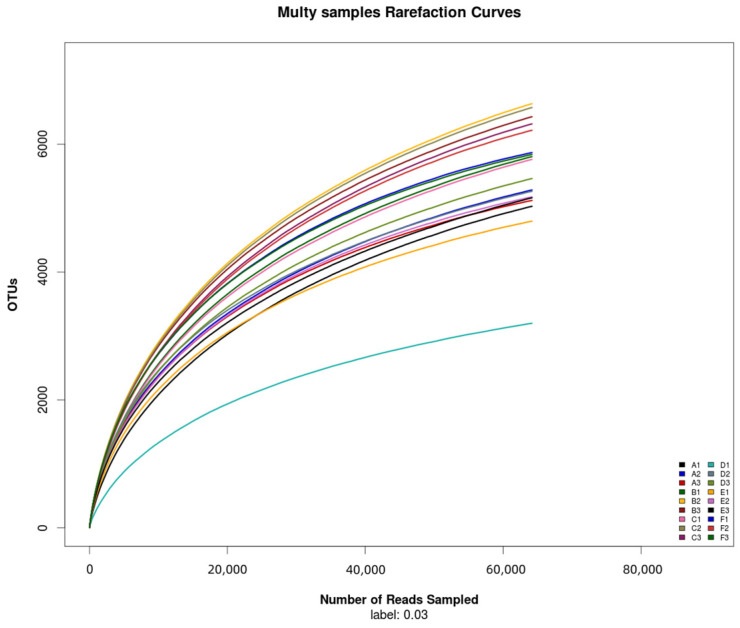
Rarefaction curves of samples.

**Figure 3:**
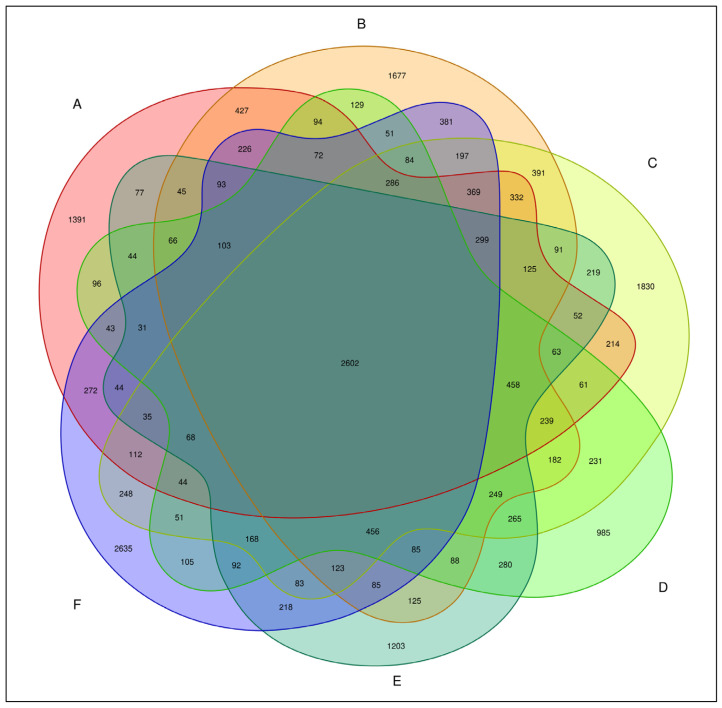
Venn graph of bacteria OTU distribution.

The total number of bacterial OTUs in melon root soil under different treatments was 2602, accounting for 7.81% of the total OTUs. The number of unique bacteria OTUs treated by CK(A), KT1(B), KT2(C), KT3(D), KT4(E), and KT5(F) was 1391, 1677, 1830, 985, 1203, and 2635, accounting for 4.18%, 5.04%, 5.41%, 2.96%, 3.61%, and 7.91% of the total OTUs (Figure 2). The OTUs were obtained by clustering high-quality sequences, which were then flattened and displayed using a Venn diagram (Figure 3). Totals of 5143, 6288, 6216, 4640, 5043, and 5974 OTUs were obtained by CK(A), KT1(B), KT2(C), KT3(D), KT4(E), and KT5(F) treatments.

The Alpha diversity analysis of soil bacteria in melon soil affected by different soil conditioner ratios in the melon rhizosphere is shown in Table 4. According to the superspecies, the bacterial flora richness index, bacterial flora index, and aroma index of the KT2 treatment were the highest among all groups, significantly increasing by 24.91%, 25.36%, and 6.75% compared to CK; the Simpson index had no significant difference among all groups. The coverage of samples was 97%, which effectively reflected the information of bacterial community diversity in soil samples.

**Figure 4:**
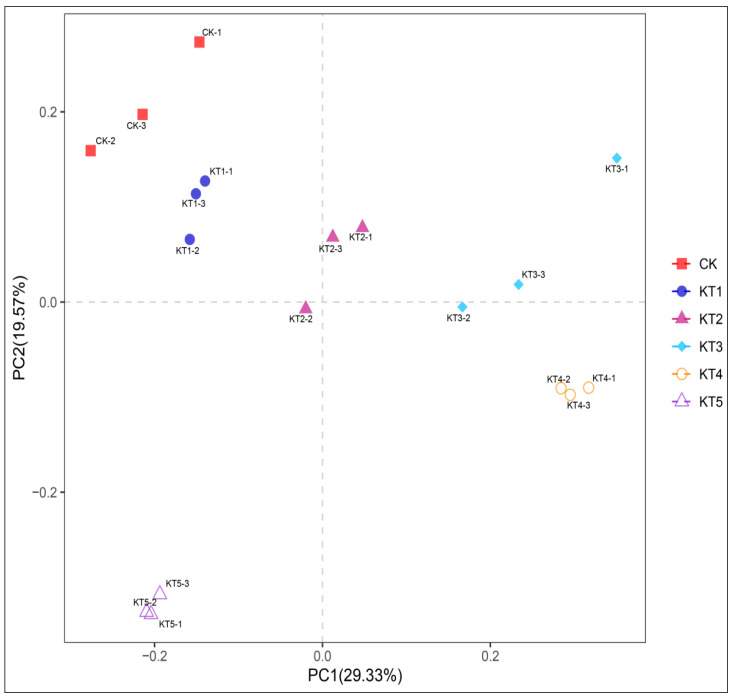
PCoA analysis of four soil sample communities.

The Principal Coordinate Analysis (PCoA) of soil bacteria in melon roots under different soil regulator ratios showed that the contribution rates of PCoA1 and PCoA2 in soil bacterial communities were 29.33% and 19.57% (Figure 4). CK had a significant distance from KT2, KT3, KT4, and KT5, indicating that the composition of the soil bacterial community was different among all groups. In particular, KT4 and CK were significantly separated in PCoA1 and PCoA2, indicating that there were differences in community structure. According to the results of β diversity analysis, the rhizosphere soil of melon treated with different soil mixing ratios affected the composition of the soil bacterial community compared to the control.

### 2.4. Effects of Different Soil Regulator Ratios on Soil Bacterial Flora Composition and Relative Abundance of Melon Roots

**Figure 5:**
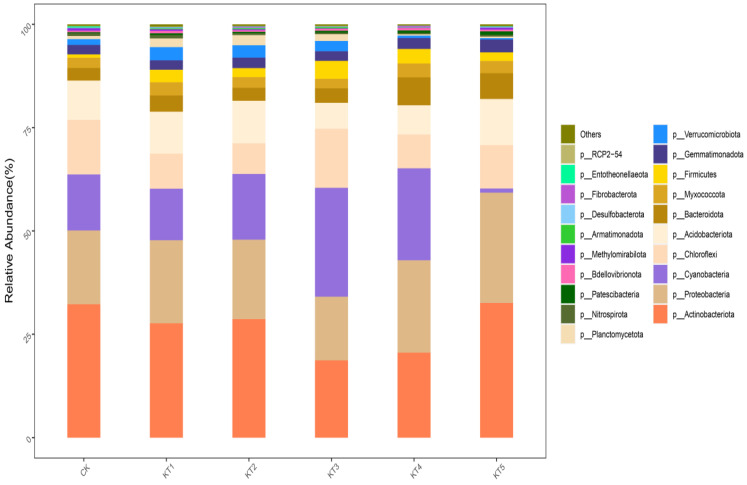
Relative abundance of bacterial communities at the phylum level.

The 33,304 OTUs in the rhizosphere soil samples of melon were divided into 48 phyla, 139 classes, 364 orders, 556 families, and 1180 genera. Based on the distribution of the proportion of dominant bacteria in each group at a phyla classification level of >1% (Figure 5), the dominant bacteria in the treated samples were mainly *Actinobacteriota*, *Proteobacteria*, *Cyanobacteria*, *Chloroflexi*, *Acidobacteria*, *Bacteroidetes*, *Myxomycota*, *Firmicutes*, *Gemmatimonadota*, *Verrucomicrobia*, and *Planctomycetes*, which accounted for 96.59~97.63% of the relative abundance of all bacterial groups.

The relative abundance of Actinomycta was significantly different among different treatments; its relative abundance in the CK, KT1, KT2, KT3, KT4, and KT5 treatments accounted for 32.27%, 27.65%, 28.65%, 18.68%, 20.56%, and 32.57%. The relative abundance of Proteobacteria in CK, KT1, KT2, KT3, KT4, and KT5 treatments was 17.84%, 20.11%, 19.23%, 15.41%, 22.35%, and 26.69%. There was no significant difference in the relative abundance of *Cyanobacteria* among all treatments; its highest value was 26.35% in KT3, while its lowest value was 1.01% in KT5. The order among groups was as follows: KT3 > KT4 > KT2 > CK > KT1 > KT5. The relative abundance of *C. viridis* was significantly different among different treatments; its relative abundance in the CK, KT1, KT2, KT3, KT4, and KT5 treatments accounted for 13.2%, 8.48%, 7.43%, 14.3%, 8.17%, and 10.49%. The relative abundance of *Acidobacteria* in the CK, KT1, KT2, KT3, KT4, and KT5 treatments was 9.52%, 10.16%, 10.29%, 6.26%, 7.08%, and 11.17%. The relative abundance of *Bacteroidetes* in CK, KT1, KT2, KT3, KT4, and KT5 treatments was 3.06%, 3.94%, 3.14%, 3.5%, 6.76%, and 6.25%. There was no significant difference in the relative abundance of *Myxomycta* among all treatments; its highest value was 3.36% in KT4, while its lowest value was 2.33% in KT3. The relative abundance of *Firmicutes* in the CK, KT1, KT2, KT3, KT4, and KT5 treatments was 0.81%, 3.03%, 2.17%, 4.33%, 3.51%, and 2.1%. There was no significant difference in the relative abundance of *Blastomonas* among all treatments; its highest value was 3.12% in KT5, while its lowest value was 2.29% in KT1. The relative abundance of *Verrucobacteria* was significantly different among different treatments; its relative abundance in the CK, KT1, KT2, KT3, KT4, and KT5 treatments was 1.41%, 3.18%, 3%, 2.48%, 0.58%, and 0.32%. The relative abundance of *Pontomyces* was significantly different among different treatments; its relative abundance in the CK, KT1, KT2, KT3, KT4, and KT5 treatments was 0.75%, 2.11%, 2.46%, 1.65%, 0.33%, and 0.37%, respectively.

### 2.5. Effects of Environmental Factors on the Distribution of Bacterial Communities in the Rhizosphere Soil of Melon

**Figure 6:**
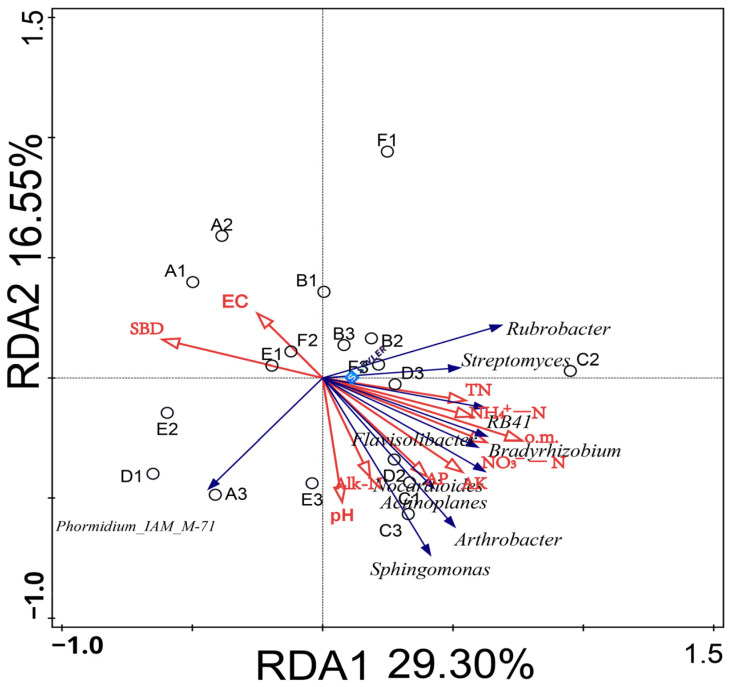
Effects of environmental factors on the distribution of bacterial communities in melon root soil. (Note: Dots represent matrix samples; the red arrow represents the physical and chemical properties of the matrix; the blue arrow represents the matrix microbe; and the angle between the influencing factors (between the factor and the sample) is an acute angle, indicating that the two factors are positively correlated. The obtuse angle is a negative correlation, whereby the longer the ray, the greater the role of the factor. EC: electrical conductivity; pH: pH; o.m.: organic matter; TN: total nitrogen; AP: available phosphorus; AK: rapidly available potassium; Alk-N: alkali-hydrolyzed nitrogen; NH^4+^-N: ammonium nitrogen; NO^3−^-N: nitrate nitrogen; and SBD: volume weight.)

As an important factor affecting the characteristics of soil bacterial flora, the relationship between soil environmental factors and bacterial communities needs to be further studied. Therefore, the 10 selected bacterial genera and 10 soil physical and chemical indexes were selected for redundancy analysis (RDA) (Figure 6). The results indicated that different soil conditioner ratios affected the distribution of bacterial communities in the soil. Among them, the RDA1 and RDA2 axes explained 29.30% and 16.55% of the variation; the total explanation rate reached 45.85%, which could better reflect the relationship between the level of bacterial genera and environmental factors in the rhizosphere soil of melon under different soil regulator ratios.

The physical and chemical properties of soil were mainly concentrated in the second and fourth quadrants. The results of replacement tests showed that the cumulative interpretation rate of o.m., EC, AP, and NO^3−^-N (*p* < 0.05) was 40.1%, which was the dominant factor of bacterial community change at the dominant genus level. Arthrobacter, Sphingosphingomonas, Bradyrhizobium, and Rubra were positively correlated with o.m., AP, and NO^3-^-N, but were negatively correlated with EC. Phormidium_IAM_M-71 was negatively correlated with o.m., EC, AP, and NO^3-^-N (Table 5).

## 3. Discussion

Some studies have shown that single and combined applications of different types of soil conditioner can improve the fruit quality of melon. For example, the increased application of earthworm manure [16], Gamao soil conditioner [17], soil conditioner No. 1 [18], and Trichoderma [19] had positive improvement effects on the vitamin C, soluble sugar, soluble protein, soluble solid matter, and organic acid contents of melon fruits (Table 2). In this study, treatment with 15% added ash soil conditioner significantly improved the fruit quality and yield of melon compared to CK, demonstrating the greatest improvement among all groups. In addition, treatment with 5% and 25% added ash soil conditioner also significantly increased the yield of melon, but less so than that of 15% added ash soil conditioner (Table 1). This is consistent with the research results of Katarzyna, C [20] and Wang, X [21].

Soil nutrients and organic matter are important prerequisites and guarantees for normal plant growth. In this study, it was found that the pH of melon rhizosphere soil was significantly improved by different amounts of added ash soil conditioner; the contents of organic matter, total nitrogen, alkali-hydrolyzed nitrogen, nitrate nitrogen, ammonium nitrogen, available potassium, and available phosphorus in melon rhizosphere soil were also significantly increased with the addition of 15% ash soil conditioner, reporting the highest contents among all treatment groups. This is consistent with the research results of Mulualem, T [22] and Shuhong, L [23]. However, the addition of 35% and 45% showed a poor performance, especially treatment 35% addition, which significantly reduced the content of ammonium nitrogen as well as the electrical conductivity and bulk density. This might be due to the addition of a large amount of volcanic ash over-absorbing soil nutrients such as nitrogen, available phosphorus, and available potassium and excessive base exchange capacity leading to the formation of soil secondary salinization, resulting in a decrease in soil water retention and air permeability as well as an increase in soil bulk density and electrical conductivity [24].

Soil microorganisms are responsible for maintaining soil vitality and play an important role in maintaining the overall service function of soil ecosystems. When the soil microbial ecological environment is disturbed, the microbial quantity, activity, diversity, and community structure will be affected [25,26]. In this study, through the analysis of the Alpha diversity of soil bacteria in melon roots, it was found that the Chao1 and ACE indexes both reached their highest values when the 15% ash soil conditioner treatment was applied, while the treatment with 35% had no significant effect compared to CK. However, the abundance of bacterial flora in the rhizosphere soil of melon was reduced to some extent. At the same time, the Shannon index was also significantly increased by the addition of 15% ash soil conditioner; the reason for this is that this method of treatment has the strongest ability to regulate the physical and chemical properties of melon rhizosphere soil, such as the greatest observed extent in the reduction in soil bulk density and electrical conductivity, which plays a regulating role in the survival mode and development metabolism of soil microorganisms [27]. Moreover, volcanic ash has a relatively large surface area and a strong electrostatic field. Its pore structure provides a basis for the survival, growth, and development of soil microorganisms. However, the treatment with the highest amount of added volcanic ash is not conducive to the growth of soil microorganisms due to the larger soil bulk density and high salinization degree; as such, the richness and diversity of the soil bacterial community are reduced.

In the investigation of bacterial community structure and relative abundance in the rhizosphere soil of melon, it was found that the application of soil conditioner did not change the dominant bacteria in the rhizosphere soil of melon. The six dominant bacteria were concentrated in *Actinomycetes*, *Proteobacteria*, *Cyanobacteria*, *Chloromycetes*, *Acidobacteria*, *Bacteroides*, *Myxomycetes*, *Firmicutes*, *Blastomonas*, *Verrucobacteria*, and *Pontomyces.* This is consistent with the research results of Shen Xiaoqing [28] and Zhang Ang [29]. However, the relative abundance of microphyla in each treatment changed significantly. Further studies found that the dominant bacterial genera of soil conditioner and control treatment were not the same. Among them, the dominant bacteria genera treated with 15% volcanic ash soil conditioner were *Flavobacteria*, *Nocardia*, *Bradyrhizobia*, *Actinomycete*, *Arthrobacter*, *Streptomyces*, *Sphingosinomonas*, and RB41. The dominant bacterium genus treated with 45% ash soil conditioner was Erythrobacterium, while the dominant bacterium in CK was Phormidium_IAM_M-71.

## 4. Materials and Methods

### 4.1. Materials

The melon variety “Da Shetou” was provided by Jilin Vegetable Research Institute (Changchun, China); mineral biological soil conditioner was jointly developed by the research group and Jilin Jiujin Agricultural Technology Co., Ltd. (Jilin, China).

Potted garden soil was obtained from Jilin Agricultural University College of Horticulture teaching test base five arch. The bulk density was 0.92 g·cm^−3^, the pH of the tested soil was 5.28, the organic matter content was 55.31 g·kg^−1^, the alkali-hydrolyzed nitrogen content was 222.78 mg·kg^−1^, the available phosphorus content was 96.05 mg·kg^−1^, and the available potassium content was 225.33 mg·kg^−1^. The pH value of the soil conditioner was 7.34, the organic matter content was 55.70%, the available phosphorus was 5.6 mg·kg^−1^, the available potassium was 159.8 mg·kg^−1^, and the total nitrogen was 0.011 g·100 g^−1^.

The daytime temperature of the greenhouse was 30~35 °C, while the night-time temperature was 15~18 °C. The relative air humidity was 60~75%.

### 4.2. Methods

The experiment was conducted in the greenhouses of Jilin Agricultural University from April to September 2023. The potted plant method (bottom diameter 23.5 cm, diameter 34 cm) with a random block experiment design was utilized. A total of 6 treatments (Table 6), with 1 plot per treatment and a plot area of 6 m^2^, resulting in 18 pots per replicate and a total of 3 replicates, were carried out; the plant spacing was 40 cm and the ridge width was 60 cm. Seeding was carried out on 24 April and planting on 21 May; double row planting, with row spacing of 160 cm × 40 cm, was used. The growth period was uniform. The first 5 leaves of the melon were cored; 1 plant was classified as 2 melons. Pollination began on 22 June, and the harvest was completed on 12 August. Other management was the same as the usual local production management.

#### 4.2.1. Melon Plant Samples and Rhizosphere Soil Sample Detection

After planting, 3 plants with uniform growth were randomly selected to be labeled for each treatment; their growth indicators were investigated every 15 days. The root shaking method and “S” shape random sampling were used. The collected rhizosphere soil of melon was screened by 2 mm to remove impurities such as roots and weeds and was then transferred to the laboratory. Soil samples for microbial qPCR analysis and high-throughput sequencing samples were stored at −80 °C, soil samples for DNA extraction and soil enzyme activity analysis were stored at 4 °C, and soil samples for physicochemical analysis were naturally air-dried at room temperature.

#### 4.2.2. Measurement of Yield and Quality Index

The average fruit weight, number of fruits, and yield per plant were calculated to convert the yield per acres. The content of vitamin C was determined using molybdenum blue colorimetry. The organic acid content was determined using acid–base titration [30]; the soluble protein content was determined using Coomassie brilliant blue colorimetry [30]; the content of soluble sugar was determined using anthrone colorimetry [30]; and the content of soluble solid was determined using an Abbe refractometer (Zhejiang, China).

#### 4.2.3. Soil Physical and Chemical Properties Testing

Total nitrogen content was determined using the Kjeldahl method; alkali-hydrolytic nitrogen was measured using the alkali diffusion method [31]; the available phosphorus was determined using sodium bicarbonate extraction and spectrophotometer colorimetry; the content of available potassium was determined using ammonium acetate extraction and a flame photometer [31]; organic matter was determined using the potassium dichromate volumetric method; and soil pH was measured using a potentiometer.

#### 4.2.4. Sequencing Sample Preparation

##### DNA Extraction, PCR Amplification, and MiSeq

Total DNA was extracted using the Omega Stool DNA Kit (MoBio Laboratories, Carlsbad, CA, USA). The DNA quality and concentration were measured using spectrophotometry. Using soil DNA as template, upstream primer 338 (5′-ACTCCTACGGGAGGCAGCAG-3′) and downstream primer 806R (5′-GGACTACNNGGGTATCTAAT-3′) were used to amplify the V3–V4 region of bacterial 16Sr RNA gene. An 8 bp barcode sequence was added to each of the 5′ ends of the upstream and downstream primers to distinguish between different samples. PCR products were detected using 1% Agarose gel electrophoresis and were purified using an Agencourt AMPure XP nucleic acid purification kit (Beckman Coulter, Bria, CA, USA). PCR products were used to construct the microbial diversity sequencing library, the Illumina MiSeq PE300 (Illumina Inc., San Diego, CA, USA) high-throughput sequencing platform was used for paired-end sequencing, and the original sequencing sequences were uploaded to the NCBI SRA database.

##### Data Analysis Processing

The disembarkation data were separated using QIIME1 (v1.8.0) software, according to Barcode sequence; Pear (v0.9.6) software was used to filter and splice the data, which involved removing scores lower than 20 as well as those containing fuzzy bases and primer mismatch sequences. When splicing, the minimum overlap was set to 10 bp and the mismatch rate was 0.1. After concatenation, Vsearch (v2.7.1) software was used to remove sequences of less than 230 bp in length, while the chimeric sequences were removed using the uchime method based on the Gold Database. The similarity threshold of sequences was 97%. To ensure that the coverage of all samples was fairly high, the data volume of all samples was homogenized to 25,323 sequences. Compared with the Silva128 database using RDP Classifier algorithm, a confidence threshold of 70% was set and the species classification information corresponding to each OTU was obtained. Based on species annotations and relative abundance results, the species composition histogram was analyzed using R (v3.6.0) software.

### 4.3. Data Processing

SPSS was used for difference significance analysis, while Vsearch (v2.7.1) software uparse algorithm was used for Operational Taxonomic Unit (OTU) clustering of high-quality sequences. Alpha diversity analysis (including the Shannon, Simpson, and Chao1 indexes) was carried out using QIIME1 (v1.8.0) software. The species composition histogram was analyzed using R (v3.6.0) software. The beta diversity distance matrix was calculated using QIIME1 (v1.8.0) and, based on Weighted UniFrac distance, cluster heat map and redundancy analyses (RDA) were performed using R (v3.6.0) software.

## 5. Conclusions

Adding an appropriate amount of soil conditioner to melon soil can promote the growth and development of melon and can effectively improve its soil environment. The main results are as follows:

1. The treatment of adding 15% Jiujin soil conditioner to melon soil had the best effect on yield and quality compared with the control treatment. However, a 35% supplementation resulted in a decrease of 6.24% in soluble solid content, 8.74% in vitamin C content, and 13.07% in solid acid ratio compared to the control, thereby inhibiting the enhancement of fruit quality.
2. The treatment of adding 15% soil conditioner to melon soil had the best improvement effect on the soil’s physical and chemical properties and enzyme activity compared with the control. The treatment with 35% added soil conditioner significantly reduced the ammonium nitrogen content of the soil by 1.62%, as well as its sucrase and catalase activity by 7.67% and 2.09%, respectively.
3. The richness, ACE, and Shannon indexes of the bacterial community in the rhizosphere soil of melon that had been treated with 15% soil conditioner were significantly higher than those of the control treatment. It also increased the relative abundance of beneficial bacteria such as *Flavobacterium*, *Actinoplanes*, *Arthrobacter*, *Streptomyces*, and *Sphingomonas* as well as improving the bacterial community in the rhizosphere of melon. RDA (redundancy analysis) found that the main influencing factors of soil bacterial community structure were organic matter, electrical conductivity, available phosphorus, and nitrate nitrogen in melon rhizosphere soil.

In summary, under the conditions of this experiment, the recommended added amount of Jiujin soil conditioner in facility melon production is 15%.

## Figures and Tables

**Table 1:** Effect of different soil conditioning agent ratios on melon yield.

Treatment	Single Fruit Weight (g)	Yield (kg/667 m^2^)
CK	383.00 ± 4.00 bc	2489.61 ± 19.38 bc
KT1	390.00 ± 6.00 b	2813.26 ± 24.66 a
KT2	399.67 ± 5.23 a	2893.92 ± 19.65 a
KT3	390.67 ± 7.23 b	2839.65 ± 23.67 a
KT4	377.33 ± 3.67 c	2469.87 ± 21.14 c
KT5	388.33 ± 5.67 b	2498.63 ± 16.77 b

Note: Different letters in the same column indicate significant differences (*p* < 0.05). Three repeats were used; the same applies below.

**Table 2:** Effect of different soil conditioning agent ratios on melon fruit quality.

Treatment	Soluble Protein (mg·100 g^−1^)	Dissolved Solid (%)	Soluble Sugar (mg·g^−1^)	Vitamin C (mg·100 g^−1^)	Titratable Acid (%)	Dissolved Solid
CK	1.74 ± 0.15 cd	14.82 ± 0.35 c	42.62 ± 1.58 bc	12.13 ± 0.67 b	0.67 ± 0.01 d	21.12 ± 1.47 b
KT1	1.79 ± 0.11 bc	14.95 ± 0.16 c	45.51 ± 1.21 ab	12.33 ± 0.08 b	0.71 ± 0.01 c	21.05 ± 2.11 b
KT2	1.83 ± 0.12 ab	15.89 ± 0.57 b	45.68 ± 0.94 a	14.13 ± 0.23 a	0.71 ± 0.02 c	22.38 ± 0.98 a
KT3	1.89 ± 0.05 a	16.35 ± 0.46 a	46.44 ± 1.27 a	13.79 ± 0.05 a	0.76 ± 0.01 b	21.51 ± 0.75 b
KT4	1.72 ± 0.01 d	13.95 ± 0.31 d	42.18 ± 0.44 c	11.07 ± 0.93 c	0.76 ± 0.01 b	18.36 ± 1.14 c
KT5	1.77 ± 0.02 bcd	14.88 ± 0.26 c	41.13 ± 1.11 c	12.52 ± 0.25 b	0.79 ± 0.03 a	18.84 ± 1.26 c

Note: Different letters in the same column indicate significant differences (*p* < 0.05). Three repeats were used; the same applies below.

**Table 3:** Effects of different soil physical and chemical conditioners on soil physical and chemical indicators.

Treatment	pH	EC (ds·m^−1^)	Volumetric Weight (g·cm^−3^)	Organic Matter (g·kg^−1^)	Total Nitrogen (g·kg^−1^)	Alkaline-Hydrolyzed Nitrogen (mg·kg^−1^)	Nitrate Nitrogen (mg·kg^−1^)	Ammonium Nitrogen (mg·kg^−1^)	Rapidly Available Potassium (mg·kg^−1^)	Available Phosphorous (mg·kg^−1^)
CK	5.28 d	0.38 a	0.93 a	55.31 d	1.04 b	222.78 b	15.35 de	7.52 c	225.33 c	96.05 bc
KT1	5.46 d	0.36 ab	0.90 b	61.71 bc	1.16 ab	245.22 ab	16.20 c	7.63 b	231.67 bc	97.25 bc
KT2	5.53 c	0.31 bc	0.86 c	66.81 a	1.28 a	276.11 a	17.07 b	7.76 a	260.67 a	112.71 a
KT3	5.52 c	0.29 c	0.83 d	64.49 ab	1.19 ab	268.34 ab	16.00 c	7.63 b	243.33 b	114.54 a
KT4	5.47 b	0.38 a	0.80 e	55.77 d	1.14 b	217.33 b	14.92 e	7.40 d	221.00 c	92.25 c
KT5	5.34 a	0.37 a	0.78 f	59.16 c	1.19 ab	226.32 ab	15.74 cd	7.55 c	226.67 c	100.56 b

Note: Different letters in the same column indicate significant differences (*p* < 0.05). Three repeats were used; the same applies below.

**Table 4:** Effect of different soil conditioners on Alpha diversity of bacterial communities in melon rhizosphere soil.

Treatments	Chao1 Index	ACE Index	Shannon Index	Simpson Index	Coverage (%)
CK	6336.72 ± 109.86 bc	6571.65 ± 248.35 c	9.78 ± 0.33 bc	0.99 ± 0.01 a	98 a
KT1	7793.17 ± 50.57 ab	6727.61 ± 165.39 bc	10.19 ± 0.17 abc	0.99 ± 0.01 a	98 a
KT2	7915.86 ± 73.28 a	8238.01 ± 196.99 a	10.44 ± 0.19 a	0.99 ± 0.00 a	98 a
KT3	6956.12 ± 97.86 abc	8169.41 ± 159.59 ab	10.31 ± 0.09 abc	0.99 ± 0.00 a	98 a
KT4	5938.36 ± 36.20 c	6146.87 ± 217.13 c	9.66 ± 0.21 c	0.99 ± 0.00 a	98 a
KT5	6399.07 ± 118.45 bc	7377.01 ± 230.99 abc	9.89 ± 0.45 bc	0.99 ± 0.01 a	98 a

Note: Different letters in the same column indicate significant differences (*p* < 0.05). Three repeats were used; the same applies below.

**Table 5:** Monte Carlo permutation test on the influencing factors of soil bacterial community.

Factors	Explanation/%	Contribution/%	R Square	*p*-Value
o.m.	20.0	32.9	16.0	0.001
EC	7.7	12.7	2.56	0.027
SBD	4.2	6.9	0.81	0.049
AP	5.8	9.6	1.44	0.042
NO^3−^-N	6.6	10.9	1.96	0.036
TN	3.2	5.3	0.49	0.058
AK	2.8	4.7	0.36	0.147
NH^4+^-N	3.3	5.4	0.36	0.216
Alk-N	5.3	8.7	1.0	0.374
pH	1.7	2.8	0.9	0.417

**Table 6:** Application ratio of soil conditioner.

Treatment	Ratio of Soil Conditioner (%)
CK (Pastoral soil)	0
KT1	95% Pastoral soil + 5% volcanic ash soil conditioner
KT2	85% Pastoral soil + 15% volcanic ash soil conditioner
KT3	75% Pastoral soil + 25% volcanic ash soil conditioner
KT4	65% Pastoral soil + 35% volcanic ash soil conditioner
KT5	55% Pastoral soil + 45% volcanic ash soil conditioner

## Data Availability

The datasets generated during and/or analyzed during the current study are available from the corresponding author on reasonable request.
